# ﻿Three new species of *Apiospora* (Amphisphaeriales, Apiosporaceae) in China

**DOI:** 10.3897/mycokeys.112.135493

**Published:** 2025-01-20

**Authors:** Kunmin Yu, Hong Zhang, Kexin Cheng, Yulan Jiang

**Affiliations:** 1 Department of Plant Pathology, College of Agriculture, Guizhou University, Guiyang, 550025, China Guizhou University Guiyang China; 2 Department of Food Science and Engineering, Moutai Institute, Renhuai, 564502, China Moutai Institute Renhuai China

**Keywords:** Ascomycota, fungal taxonomy, morphology, novel species, phylogeny

## Abstract

China is located in eastern Asia and has a large quantity and rich variety of bamboo resources. Bamboo resources also contain various fungal species such as *Apiospora.* The genus *Apiospora* is commonly used as plant pathogens, endophytes, and saprophytes, which are widely present in various host ranges around the world. The discovery of metabolites has been proven to play an important role in the pharmaceutical industry. Recently, 10 strains were isolated from bamboo in Guizhou Province, China, which were identified as five species of *Apiospora* based on multigene phylogenetic analysis of ITS, LSU, *TEF1-α*, and *TUB2*, and morphological observations, including two recognized species, *Ap.arundinis* and *Ap.lophatheri*, and three novel species, viz. *Ap.bambusicaulis*, *Ap.bambusirimae*, and *Ap.bambusilentiginis*. Illustrations and descriptions of these taxa are provided.

## ﻿Introduction

The *Apiospora* is a large genus of the family Apiosporaceae (Amphisphaeriales, Sordariomycetes, Ascomycota) (Hyde et al. 2020; Pintos and Alvarado 2021), with diverse species worldwide, in various climates, primarily in temperate and tropical regions. These fungi have been found in different substrates, including plants, air, soil, freshwater, lichens, marine environments, insect intestines, and human tissues (Tian et al. 2021). Most *Apiospora* species are found as saprophytes and endophytes of many plant hosts; several species have been reported as important plant pathogens (Wang et al. 2018; Kwon et al. 2022), such as *Ap.kogelbergense* and *Ap.sacchari* that cause *Bambusaintermedia* and *Triticumdurum* wilt disease and *Ap.xencodella* that causes *Pistaciavera* fruit wilt disease (Mavragani et al. 2007; Aiello et al. 2018; Yin et al. 2021). Additionally, some *Apiospora* species have been sources of bioactive compounds, industrial enzymes, and antifungal agents, with enormous commercial potential in the pharmaceutical industry (Shrestha et al. 2015; Heo et al. 2018).

*Apiospora* was introduced by Saccardo (1875) with *Ap.montagnei* as the type species. The multi-locular perithecial stromata with hyaline ascospores surrounded by a thick gelatinous sheath is the characteristic of sexual morphs of *Apiospora*. (Dai et al. 2017; Pintos and Alvarado 2021). *Apiospora* was treated as the sexual morph of *Arthrinium* for years because of their similar asexual morphological characters, especially their same basauxic conidiogenesis (von Höhnel 1919; Petrak 1925; Hudson 1960, 1963; Ellis 1965; Samuels et al. 1981). Hyde et al. (1998) established the family Apiosporaceae to accommodate genera with basauxic conidiogenesis, and *Apiospora* was placed inside. Subsequently, *Arthrinium* and *Apiospora* were formally synonymized based on phylogenetic analyses of ITS, *TEF1-α*, and *TUB2* sequence data by Crous and Groenewald in 2013, and they emphasized that most species of *Arthrinium* (= *Apiospora*) recorded in the Poaceae hosts and some were collected from the Cyperaceae and Juncaceae hosts. Later, Wang et al. (2018) observed two species, *Ar.japonicum* and *Ar.puccinioides*, occurring on *Carex* spp. of the Cyperaceae, and Pintos et al. (2019) published the genetic data of *Ar.cariciola* (the type species of *Arthrinium*), *Ar.curvatum*, and *Ar.sporophleum* found in *Carex* and *Juncus* of the Juncaceae, and these samples also formed an independent clade, obviously unrelated with other data of *Arthrinium* (= *Apiospora*). In addition, these authors found that the data from Cyperaceae and Juncaceae mainly occurred in temperate, cold, or alpine regions, and these species often produce rounded, fusoid, navicular, polygonal, or curved conidia, while other data of *Arthrinium* (= *Apiospora*) mainly occur in the Poaceae family in a wide range of habitats, including tropical and subtropical regions (there are also other plants), and they have rounded, lenticular conidia (Hudson 1976; Pintos et al. 2019; Pintos and Alvarado 2021). On these foundations, Pintos and Alvarado (2021) selected the type strain *Ap.montagneiand* separated *Apiospora* and *Arthrinium* as two distinct genera according to ITS, LSU, *TEF1-α*, and *TUB2* sequence data. The third group of species previously classified as *Arthrinium*, including *Ar.urtiaca* M.B. Ellis (1965) and *Ar.trachycarpi* C.M. Tian & H. Yan (Yan et al. 2024), were likely not related to *Apiospora* or *Arthrinium* (Tang et al. 2021), and therefore Jiang et al. (2022) proposed the new Arthrinium-like genus *Neoarthrinium* in Amphisphaeriales to accommodate them.

It is extremely difficult to distinguish asexual morphs of these three genera based on morphological characters alone. Therefore, molecular phylogenetic information is very important to accurately distinguish them (Pintos and Alvarado 2021; Jiang et al. 2022). In this study, ten strains were collected from bamboo in Guizhou Province, China, which were identified as two known species and three new species based on morphological characteristics and multigene phylogenetic analysis.

## ﻿Materials and methods

### ﻿Sample collection and fungal isolation

Diseased bamboo branches were observed and collected from Huaxi District, Guiyang City, Guizhou Province, China. The samples were placed in clean plastic bags and transported to the laboratory for isolation. These strains were isolated and purified using the method proposed by Xie et al. (2024) to obtain pure colonies. The re-isolated and purified fungi were placed on potato glucose agar (PDA) plates and incubated for 5–10 days at 25 °C in the dark. The holotype and ex-type cultures are deposited in the Culture Collection of the Department of Plant Pathology, Agriculture College, Guizhou University, China (**GUCC**).

### ﻿Morphological examination

Morphological characteristics were based on fertile cultures grown on PDA in a constant temperature incubator at 25 °C. Colony diameter and characteristics were recorded after 5–7 days. Colony colors (surface and reverse) were recorded using the color charts of Rayner (1970). Morphological descriptions were based on cultures sporulating on water agar (WA) and using 15–30% lactic acid on a glass slide. The size of the conidia and conidiogenous cells was shown as minimum-maximum. Following spore production, observations were made with a Zeiss Axioscope compound microscope equipped with differential interference contrast (DIC). Morphological indicators of 30 conidiogenous cells and conidia were measured using Image Frame Work (IFW) software. The taxonomic information of the new taxa was deposited in MycoBank (http://www.mycobank.org).

### ﻿DNA extraction and PCR amplification

Total genomic DNA was extracted from mature mycelium grown on PDA using the BIOMIGA Fungus Genomic DNA Extraction Kit GD2416 (Biomiga, CA, USA), performed following the manufacturer’s protocol. The DNA was amplified and sequenced by polymerase chain reaction (PCR). DNA sequences of four different loci were obtained, including the nrDNA internal transcribed spacer regions 1 and 2 with the intervening 5.8S subunit (ITS), a partial sequence of the large subunit nrDNA subunit (LSU), a partial sequence of the translation elongation factor 1-alpha gene (*TEF1-α*), and a partial sequence of the beta-tubulin gene (*TUB2*). They were all amplified with the primer pairs and PCR program listed in Table 1. The amplified PCR products were sent to a commercial sequencing provider (Sangon Biotech, Shanghai, China) Co., Ltd. for DNA sequencing.

**Table 1.** Gene regions and respective primer pairs used in the study.

Locus	PCR primers	PCR: thermal cycles: (Annealing temperature in bold)	Reference
ITS	ITS5/ITS4	(94 °C: 30 s, 55 °C: 30 s, 72 °C: 45 s) × 29 cycles	White et al. 1990
LSU	LR0R/LR5	(94 °C: 30 s, 48 °C: 50 s, 72 °C: 1 min 30 s) × 35 cycles	Vilgalys and Hester 1990; Cubeta et al. 1991
*TEF1-α*	EF1-728F/EF2	(95 °C: 30 s, 51 °C: 30 s, 72 °C: 1 min) × 35 cycles	O’Donnell et al. 1998; Carbone and Kohn 1999
*TUB2*	Bt-2a/Bt-2b	(95 °C: 30 s, 56 °C: 30 s, 72 °C: 1 min) × 35 cycles	Glass and Donaldson 1995

### ﻿Phylogenetic analysis

The quality of the chromatograms was verified, and nucleotide sequences were assembled using SeqMan v.7.1.0. Reference sequences were obtained from previous studies (Samarakoon et al. 2022; Liu et al. 2023) and retrieved from the National Center for Biotechnology Information. The sequences were aligned using MAFFT on the web portal (Katoh et al. 2019). The sequence ends were trimmed manually to remove low-quality bases using BioEdit 7.1.3.0 (Katoh et al. 2019) and assembled using MEGA X (Kumar et al. 2018). Multi-gene phylogenetic analyses based on the combined ITS, LSU, *TEF1-α*, and *TUB2* were carried out to clarify the phylogenetic relationships of *Apiospora* species using the maximum likelihood (ML) and Bayesian inference (BI) analyses. The ML was implemented on the CIPRES Science Gateway portal (https://www.phylo.org) using RAxML-HPC BlackBox 8.2.10 (Stamatakis 2014), employing a GTRGAMMA substitution model with 1000 bootstrap replicates. The Bayesian posterior probabilities (BPP) were determined by Markov Chain Monte Carlo (MCMC) sampling in MrBayes v.3.2.6 (Ronquist et al. 2012). The six simultaneous Markov chains were run for 1 M generations, starting from random trees and sampling trees every 100^th^ generation, and 25% of aging samples were discarded, running until the average standard deviation of the split frequencies dropped below 0.01. The phylogram was visualized in FigTree v.1.3.1 (Rambaut 2012) and edited in Adobe Illustrator CS5 (Adobe Systems Inc., USA). The newly generated nucleotide sequences were deposited in GenBank (Table 2).

**Table 2.** Isolates and GenBank accession numbers used in the phylogenetic analyses.

Species	Isolate/Strain	Host/ Substrate	Origin	GenBank Accession No.			
				ITS	LSU	*TEF1-α*	*TUB2*
*Apiosporaacutiapica*	KUMCC 20-0210-T	*Bambusabambos*	China	MT946343	MT946339	MT947360	MT947366
*Ap.agari*	KUC 21333-T	*Agarumcribrosum*	Korea	MH498520	N/A	MH544663	MH498478
*Ap.aquatica*	S-642	Submerged wood	China	MK828608	MK835806	N/A	N/A
*Ap.arctoscopi*	KUC 21331-T	Egg of *Arctoscopusjaponicus*	Korea	MH498529	N/A	MN868918	MH498487
*Ap.armeniaca*	SAUCC DL1831-T	*Prunusarmeniaca*	China	OQ592540	OQ615269	OQ613313	OQ613285
	SAUCC DL1844	*Prunusarmeniaca*	China	OQ592539	OQ615268	OQ613312	OQ613284
*Ap.arundinis*	CBS 133509	*Aspergillusflavus*	USA	KF144886	KF144930	KF145018	KF144976
	CBS 44992	Egg of *Arctoscopusjaponicus*	USA	KF144887	KF144931	KF145019	KF144977
	**GUCC6.1**	**Bamboo**	**China**	**PP959159**	**PP959169**	**PP998082**	**PP998092**
	**GUCC6.2**	**Bamboo**	**China**	**PP959160**	**PP959170**	**PP998083**	**PP998093**
*Ap.babylonica*	SAUCC DL1841-T	*Salixbabylonica*	China	OQ592538	OQ615267	OQ613311	OQ613283
	SAUCC DL1864	*Saprophytic* leaves	China	OQ592537	OQ615266	OQ613310	OQ613282
*Ap.aurea*	CBS 24483-T	Air	Spain	AB220251	KF144935	KF145023	KF144981
*Ap.balearica*	CBS 145129-T	Poaceae	Spain	MK014869	MK014836	MK017946	MK017975
*Ap.bambusae*	ICMP 6889-T	Egg of *Arctoscopusjaponicus*	China	MK014874	MK014841	MK017951	MK017980
	CBS 145133	*PhyllostachysAurea*	Spain	MK014875	MK014842	MK017952	MK017981
***Ap.bambusicaulis***	**GUCC17.41**-T	**Bamboo**	**China**	**PP959151**	**PP959161**	**PP998074**	**PP998084**
	**GUCC17.42**	**Bamboo**	**China**	**PP959152**	**PP959162**	**PP998075**	**PP998085**
*Ap.bambusicola*	MFLUCC 20-0144-T	*Schizostachyumbrachycladum*	Thailand	MW173030	MW173087	MW183262	N/A
***Ap.bambusilentiginis***	**GUCC18.51-T**	**Bamboo**	**China**	**PP959155**	**PP959165**	**PP998078**	**PP998088**
	**GUCC18.52**	**Bamboo**	**China**	**PP959156**	**PP959166**	**PP998079**	**PP998089**
***Ap.bambusirimae***	**GUCC12.51-T**	**Bamboo**	**China**	**PP959153**	**PP959163**	**PP998076**	**PP998086**
	**GUCC12.52**	**Bamboo**	**China**	**PP959154**	**PP959164**	**PP998077**	**PP998087**
*Ap.biserialis*	CGMCC 320135-T	Bamboo	China	MW481708	MW478885	MW522938	MW522955
	GZCC 20-0099	Dead culms of bamboo	China	MW481709	MW478886	MW522939	MW522956
*Ap.bawanglingensis*	SAUCC BW0444-T	*Indocalamuslongiauritus*	China	OR739429	OR739570	OR753446	OR757126
	SAUCC BW0441	*Indocalamuslongiauritus*	China	OQ592551	OQ615280	OQ613324	OQ613302
*Ap.camelliae-sinensis*	LC 5007-T	*Camelliasinensis*	China	KY494704	KY494780	KY705103	KY705173
	LC 8181	*Brassicarapa*	China	KY494761	KY494837	KY705157	KY705229
*Ap.cannae*	ZHKUCC 22-0139	*Canna* sp.	China	OR164902.1	OR164949.1	OR166286.1	OR166322.1
*Ap.chiangraiense*	MFLUCC 21-0053-T	Bamboo	Thailand	MZ542520	MZ542524	N/A	MZ546409
*Ap.chromolaenae*	MFLUCC 17-1505-T	*Chromolaenaodorata*	Thailand	MT040106	MT214436	MT235802	N/A
*Ap.cordyline*	GUCC 10026	*Cordylinefruticosa*	China	MT040105	N/A	MT040126	MT040147
*Ap.coryli*	CFCC 58978 -T	*Corylusyunnanensis*	China	OR125564	OR133586	OR139974	OR139978
	CFCC 58979	*Corylusyunnanensis*	China	OR125565	OR133587	OR139975	OR139979
*Ap.cyclobalanopsidis*	CGMCC 320136-T	*Cyclobalanopsidisglauca*	China	MW481713	MW478892	MW522945	MW522962
*Ap.descalsii*	CBS 145130-T	*Ampelodesmosmauritanicus*	Spain	MK014870	MK014837	MK017947	MK017976
*Ap.dichotomanthi*	LC 4950-T	*Dichotomanthustristaniaecarpa*	China	KY494697	KY494773	KY705096	KY705167
	CGMCC 38332	*Dichotomanthestristaniicarpa*	China	KY494755	KY494831	KY705151	KY705223
*Ap.adinandrae*	SAUCC 1282B-1-T	*Adinandraglischroloma*	China	OR739431	OR739572	OR753448	OR757128
	SAUCC 1282B-2	*Adinandraglischroloma*	China	OR739432	OR739573	OR753449	OR757129
*Ap.dongyingensis*	SAUCC 0302-T	Bamboo	China	OP563375	OP572424	OP573264	OP573270
*Ap.esporlensis*	CBS 145136-T	*Phyllostachysaurea*	Spain	MK014878	MK014845	MK017954	MK017983
*Ap.euphorbiae*	IMI 285638b	*Bambusa*	Bangladesh	AB220241	AB220335	N/A	AB220288
*Ap.fermenti*	KUC 21289-T	Seaweed	Korea	MF615226	N/A	MH544667	MF615231
*Ap.gaoyouensis*	CFCC 52301-T	*Phragmitesaustralis*	China	MH197124	N/A	MH236793	MH236789
	CFCC 52302	*Phragmitesaustralis*	China	MH197125	N/A	MH236794	MH236790
*Ap.garethjonesii*	JHB004-T	Bamboo	China	KY356086	KY356091	N/A	N/A
*Ap.gelatinosa*	KHAS 11962-T	Bamboo	China	MW481706	MW478888	MW522941	MW522958
*Ap.guiyangensis*	HKAS 102403-T	Poaceae	China	MW240647	MW240577	MW759535	MW775604
*Ap.guizhouensis*	LC 5318	Air	China	KY494708	KY494784	KY705107	KY705177
	LC 5322-T	Air in karst cave	China	KY494709	KY494785	KY705108	KY705178
*Ap.hainanensis*	SAUCC 1681-T	Bamboo	China	OP563373	OP572422	OP573262	OP573268
*Ap.hispanicum*	IMI 326877-T	Maritime sand	Spain	AB220242	AB220336	N/A	AB220289
*Ap.hydei*	CBS 114990-T	*Bambusatuldoides*	China	KF144890	KF144936	KF145024	KF144982
	LC 7103	Bamboo	China	KY494715	KY494791	KY705114	KY705183
*Ap.hyphopodii*	MFLUCC 15-0003-T	Bamboo	China	KR069110	N/A	N/A	N/A
	JHB003	Bamboo	China	KY356088	KY356093	N/A	N/A
*Ap.ibericum*	AP 10118-T	*Arundodonax*	Portugal	MK014879	MK014846	MK017955	MK017984
*Ap.intestini*	CBS 135835-T	Gut of grasshopper	India	KR011352	KR149063	KR011351	KR011350
	MFLUCC 21-0045	Dead culms of bamboo	Thailand	MZ542521	MZ542525	MZ546406	MZ546410
*Ap.hysterina*	ICPM 6889 -T	Bamboo	New Zealand	MK014874	MK014841	MK017951	MK017980
	GUCC-19-5271	Faba Bean	China	OQ236601	OQ231496	OQ270096	OQ249633
	KUC21438	*Phyllostachysbambusoides* branch	South Korea	ON764019	ON787758	ON806623	ON806633
*Ap.italica*	CBS 145138-T	*Arundodonax*	India	MK014880	MK014847	MK017956	MK017985
	CBS 145139	*Arundodonax*	India	MK014881	MK014848	MK017957	MK017986
*Ap.jatrophae*	AMH-9556	*Jatrophapodagrica*	India	HE981191	N/A	N/A	N/A
	AMH-9557-T	*Jatrophapodagrica*	India	NR_154675	N/A	N/A	N/A
*Ap.jinanensis*	SAUCC DL1981-T	Bambusaceae sp.	China	OQ592544	OQ615273	OQ613317	OQ613289
	SAUCC DL2000	Bambusaceae sp.	China	OQ592543	OQ615272	OQ613316	OQ613288
*Ap.jiangxiensis*	LC 4494	*Phyllostachys* sp.	China	KY494690	KY494766	KY705089	KY705160
	LC 4577-T	*Maesa* sp.	China	KY494693	KY494769	KY705092	KY705163
*Ap.kogelbergensis*	CBS 113332	Restionaceae	South Africa	KF144891	KF144937	KF145025	KF144983
	CBS 113333-T	Restionaceae	South Africa	KF144892	KF144938	KF145026	KF144984
*Ap.koreanum*	KUC 21332-T	Egg of *Arctoscopusjaponicus*	Korea	MH498524	MH498444	MH544664	MH498482
*Ap.lageniformis*	KUC21687	*Phyllostachysnigra*	Korea	ON764023	ON787762	ON806627	ON806637
	KUC21686-T	*Phyllostachysnigra*	Korea	ON764020	ON787759	ON806624	ON806634
*Ap.locuta-pollinis*	SICAUCC 22-0036	Unknown		ON228609	ON228665	ON324018	ON237657
	LC 11683-T	*Brassicacampestris*	China	MF939595	N/A	MF939616	MF939622
*Ap.longistroma*	MFLUCC 11-0481 -T	Bamboo	Thailand	KU940141	KU863129	N/A	N/A
*Ap.lophather*i	CFCC 58975-T	*Lophatherumgracile*	China	OR125566	OR133588	OR139970	OR139980
	CFCC 58976	*Lophatherumgracile*	China	OR125567	OR133589	OR139971	OR139981
	**GUCC21.11**	**Bamboo**	**China**	**PP959157**	**PP959167**	**PP998080**	**PP998090**
	**GUCC21.12**	**Bamboo**	**China**	**PP959158**	**PP959168**	**PP998081**	**PP998091**
*Ap.machili*	SAUCC 1175A-4	*Machilusnanmu*	China	OR739433	OR739574	OR753450	OR757130
	SAUCC 1175	*Machilusnanmu*	China	OQ592560	OQ615289	OQ613333	OQ613307
*Ap.malaysiana*	CBS 102053-T	*Macarangahullettii* stem colonised by ants	Malaysia	KF144896	KF144942	KF145030	KF144988
*Ap.marianiae*	AP301119	*Phleumpratense*	Spain	ON692407	ON692423	ON677181	ON677187
*Ap.marii*	CBS 20057	*Beta vulgaris*	Netherlands	KF144900	KF144946	KF145034	KF144992
	CBS 49790-T	Atmosphere, pharmaceutical excipients, home dust and beach sands	Spain	AB220252	KF144947	KF145035	KF144993
*Ap.marinum*	KUC 21328-T	Seaweed	China	MH498538	MH498458	MH544669	MH498496
*Ap.mediterranea*	IMI 326875-T	Air	Spain	AB220243	AB220337	N/A	AB220290
*Ap.minutisporum*	17E-042-T	Soil	Korea	LC517882	N/A	LC518889	LC518888
*Ap.montagnei*	AP 301120-T	*Arundomicrantha*	Spain	ON692408	ON692424	ON677182	ON677188
*Ap.mori*	NCYUCC 19-0340	*Morusaustralis*	China	MW114314	MW114394	N/A	N/A
	MFLUCC 20-0181-T	*Morusaustralis*	China	MW114313	MW114393	N/A	N/A
*Ap.mukdahanensis*	MFLUCC 22-0056-T	Bambusoideae	Thailand	OP377735	OP377742	OP381089	N/A
*Ap.multiloculata*	MFLUCC 21-0023-T	*Bambusae*	Thailand	OL873137	OL873138	N/A	OL874718
*Ap.mytilomorpha*	DAOM 214595-T	*Andropogon*	India	KY494685	N/A	N/A	N/A
*Ap.neobambusae*	LC 7106-T	Bamboo	China	KY494718	KY494794	KY806204	KY705186
	LC 7124	Bamboo	China	KY494727	KY494803	KY806206	KY705195
*Ap.neochinensis*	CFCC 53036-T	*Fargesiaqinlingensis*	China	MK819291	N/A	MK818545	MK818547
	CFCC 53037	*Fargesiaqinlingensis*	China	MK819292	N/A	MK818546	MK818548
*Ap.neogarethjonesii*	KUMCC 18-0192-T	*Bambusae*	China	MK070897	MK070898	N/A	N/A
*Ap.neosubglobosa*	JHB006	Bamboo	China	KY356089	KY356094	N/A	N/A
	JHB007-T	Bamboo	China	KY356090	KY356095	N/A	N/A
*Ap.obovata*	LC 4940	*Lithocarpus* sp.	China	KY494696	KY494772	KY705095	KY705166
*Ap.ovata*	CBS 115042-T	*Arundinariahindsii*	China	KF144903	KF144950	KF145037	KF144995
*Ap.oenotherae*	CFCC 58972 -T	*Lophatherumgracile*	China	OR125568	OR133590	OR139972	OR139982
	LS 395	*Lophatherumgracile*	China	OR125569	OR133591	OR139973	OR139983
*Ap.paraphaeosperma*	MFLUCC 13-0644-T	Dead clumps of *Bambusa* sp.	Thailand	KX822128	KX822124	N/A	N/A
*Ap.phragmitis*	CPC 18900-T	*Phragmitesaustralis*	Italy	KF144909	KF144956	KF145043	KF145001
*Ap.phyllostachydis*	MFLUCC 18-1101-T	*Phyllostachysheteroclada*	China	MK351842	MH368077	MK340918	MK291949
*Ap.piptatheri*	AP4817A	*Piptatherummiliaceum*	Spain	MK014893	MK014860	MK017969	N/A
*Ap.pseudomarii*	GUCC 10228 -T	*Aristolochiadebilis*	China	MT040124	N/A	MT040145	MT040166
*Ap.pseudohyphopodii*	KUC 21680-T	*Phyllostachyspubescens*	Korea	ON764026	ON787765	ON806630	ON806640
	KUC21684	*Phyllostachyspubescens*	Korea	ON764027	ON787766	ON806631	ON806641
*Ap.pseudoparenchymatica*	LC 7234-T	Bamboo	China	KY494743	KY494819	KY705139	KY705211
	LC 8173	Bamboo	China	KY494753	KY494829	KY705149	KY705221
*Ap.pseudorasikravindrae*	KUMCC 20-0208-T	*Bambusadolichoclada*	China	MT946344	N/A	MT947361	MT947367
*Ap.pseudosinensis*	CPC 21546-T	Bamboo	Netherlands	KF144910	KF144957	KF145044	N/A
*Ap.pseudospegazzinii*	CBS 102052-T	*Macarangahullettii*	Malaysia	KF144911	KF144958	KF145045	KF145002
*Ap.pterosperma*	CBS 123185	*Lepidospermagladiatum*	Australia	KF144912	KF144959	N/A	KF145003
	CPC 20193-T	*Lepidospermagladiatum*	Australia	KF144913	KF144960	KF145046	KF145004
*Ap.pusillisperma*	KUC 21321-T	Seaweed	Korea	MH498533	N/A	MN868930	MH498491
*Ap.qinlingense*	CFCC 52303-T	*Fargesiaqinlingensis*	China	MH197120	N/A	MH236795	MH236791
	CFCC 52304	*Fargesiaqinlingensis*	China	MH197121	N/A	MH236796	MH236792
*Ap.rasikravindrae*	NFCCI 2144-T	Soil in karst cave	China	JF326454	N/A	N/A	N/A
*Ap.sacchari*	CBS 37267	Soil	Netherlands	KF144918	KF144964	KF145049	KF145007
	CBS 66474	Soil	Netherlands	KF144919	KF144965	KF145050	KF145008
*Ap.saccharicola*	CBS 19173	Air	Netherlands	KF144920	KF144966	KF145051	KF145009
	CBS 8317	Air	Netherlands	KF144922	KF144969	KF145054	KF145012
*Ap.sargassi*	KUC 21228-T	*Sargassumfulvellum*	Korea	KT207746	N/A	MH544677	KT207644
*Ap.sasae*	CBS 146808-T	*Sasaveitchii*	Netherlands	MW883402	MW883797	MW890104	MW890120
*Ap.septata*	CGMCC 320134-T	Bamboo	China	MW481711	MW478890	MW522943	MW522960
*Ap.serenensis*	IMI 326869-T	Food, pharmaceutical excipients, atmosphere and home dust	Spain	AB220250	AB220344	N/A	AB220297
*Ap.setariae*	CFCC 54041-T	*Setariaviridis*	China	MT492004	N/A	MW118456	MT497466
*Ap.setostroma*	KUMCC 19-0217-T	Bambusoideae	China	MN528012	MN528011	MN527357	N/A
*Ap.sichuanensis*	HKAS 107008-T	Poaceae	China	MW240648	MW240578	MW759536	MW775605
*Ap.sorghi*	URM 93000-T	*Sorghumbicolor*	Brazil	MK371706	N/A	N/A	MK348526
*Ap.sphaerosperma*	CBS114314-T	*Hordeumvulgare*	Iran	KF144904	KF144951	KF145038	KF144996
*Ap.stipae*	CBS 146804-T	*Stipagigantea*	Spain	MW883403	MW883798	MW890082	MW890121
*Ap.subglobosa*	MFLUCC 11-0397-T	Bamboo	Thailand	KR069112	KR069113	N/A	N/A
*Ap.subrosea*	LC 7291	Bamboo	China	KY494751	KY494827	KY705147	KY705219
	LC 7292-T	Bamboo	China	KY494752	KY494828	KY705148	KY705220
*Ap.taeanensis*	KUC 21322-T	Seaweed	Korea	MH498515	MH498435	MH544662	MH498473
*Ap.thailandica*	MFLUCC 15-1999	Bamboo	Thailand	KU940146	KU863134	N/A	N/A
	MFLUCC 15-0202-T	Rotten wood	China	KU940145	KU863133	N/A	N/A
*Ap.tropica*	MFLUCC 21–0056-T	Unknown	Thailand	OK491657	OK491653	N/A	OK560922
*Ap.vietnamense*	IMI 99670-T	*Citrussinensis*	Vietnam	KX986096	KX986111	N/A	KY019466
*Ap.xenocordella*	CBS 47886-T	Soil from roadway	Zimbabwe	KF144925	KF144970	KF145055	KF145013
	CBS 59566	Soil from roadway	Zimbabwe	KF144926	KF144971	N/A	N/A
*Ap.yunnana*	MFLUCC 15-0002-T	Bamboo	China	KU940147	KU863135	N/A	N/A
*Arthriniumcaricicola*	CBS 145127	*Carexericetorum*	China	MK014871	MK014838	MK017948	MK017977

Notes: Strains in this study are marked in bold. N/A = not available.

## ﻿Results

### ﻿Phylogenetic analyses

**Figure 1.**
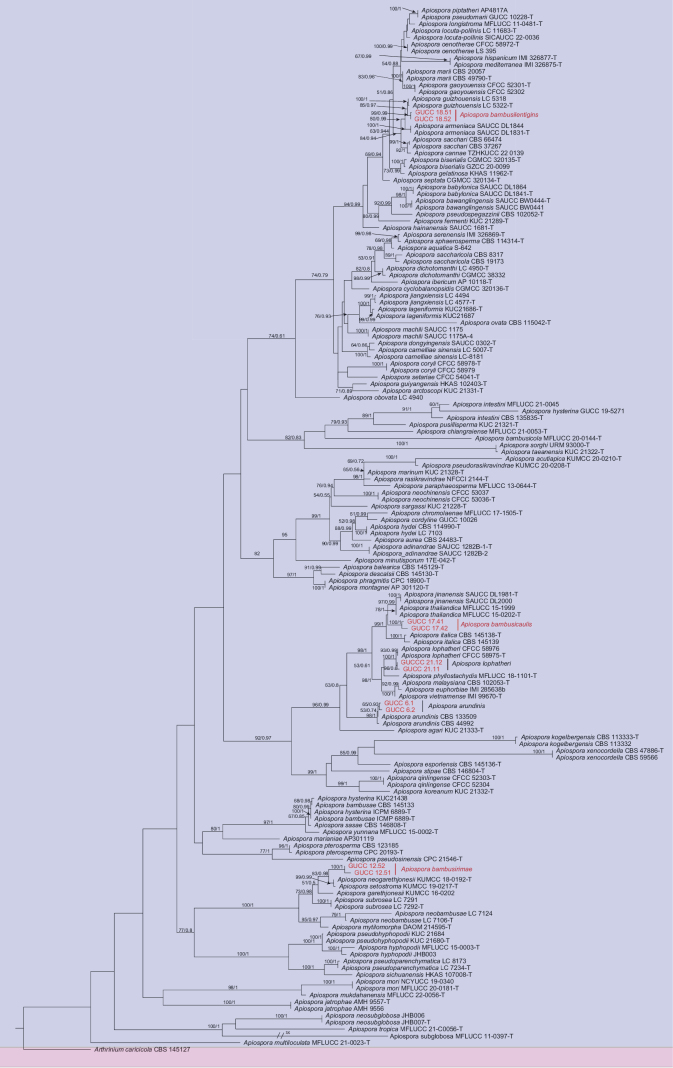
Phylogenetic tree of *Apiospora*, inferred from combined ITS, LSU, *TEF1-α*, and *TUB2* genes. *Arthriniumcaricicola* was used as an outgroup. ML bootstrap support values (≥ 50%) and Bayesian posterior probability (≥ 0.90) are shown at nodes. Strains in this study are shown in red, and type strains are marked by a “T”.

The combined ITS, LSU, *TEF1-α*, and *TUB2* sequence datasets were analyzed to infer the phylogenetic position of our ten newly sequenced strains within *Apiospora*. The dataset consisted of 160 sequences, with *Arthriniumcaricicola* (CBS 145127) as the outgroup taxon. Phylogenetic trees in this study were constructed using maximum likelihood (ML) and Bayesian inference (BI) via the CIPRES web portal with 1,000 bootstrap replicates, yielding the best ML tree (Fig. 1) with the likelihood value of –27,589.015487 and the following model parameters: Estimated base frequencies were A = 0.233824, C = 0.249823, G = 0.258804, and T = 0.257550; substitution rates were AC = 1.217800, AG = 3.016069, AT = 1.114592, CG = 0.911613, CT = 4.839047, and GT = 1.0; gamma distribution shape parameter: *α* = 0.724729. In addition, the RAxML and Bayesian analyses yielded a similar tree topology, and therefore, only the ML tree is presented (Fig. 1). The newly generated ten sequences in the present work are separated into three clades. Multi-locus phylogenetic analyses support the recognition of *Ap.arundinis* and *Ap.lophatheri* as known species and *Ap.bambusicaulis*, *Ap.bambusirimae*, and *Ap.bambusilentiginis* as newly discovered independent species (Fig. 1).

### ﻿Taxonomy

#### ﻿ Apiospora arundinis

Taxon classificationFungiAmphisphaerialesApiosporaceae

(Corda) Pintos & P. Alvarado, Fungal Syst. Evol. 7: 205 (2021)

BABCC21B-F99F-58DA-9A3B-7381DB7B1051

Fig. 2

##### Description.

***Asexual morph***: On WA, hyphae smooth, branched, septate, 1.2–3.5 µm diam. (n = 30). ***Conidiophores*** cylindrical, septate, erect, sometimes reduced to conidiogenous cells. ***Conidiogenous cells*** erect, subglobose to ampulliform, aggregated in clusters on hyphae, smooth, branched, 5–15 × 1–2.5 µm (x = 8.5 × 6 µm, n = 30). ***Conidia*** globose, sub-globose to ampulliform, lenticular, occasionally elongated to ellipsoidal, with a longitudinal germ slit, brown to dark brown, smooth to finely roughened, 6–14 × 4–7 µm (x = 10.5 × 6 µm, n = 30) µm. ***Sexual morph***: Not observed.

##### Culture characteristics.

***Colonies*** on PDA attaining 5 cm diam, after 4 days at 25 °C, thick, dense, surface with patches of grey aerial mycelia, margin irregular and undulate, diffuse yellow pigment, reverse yellow.

**Figure 2.**
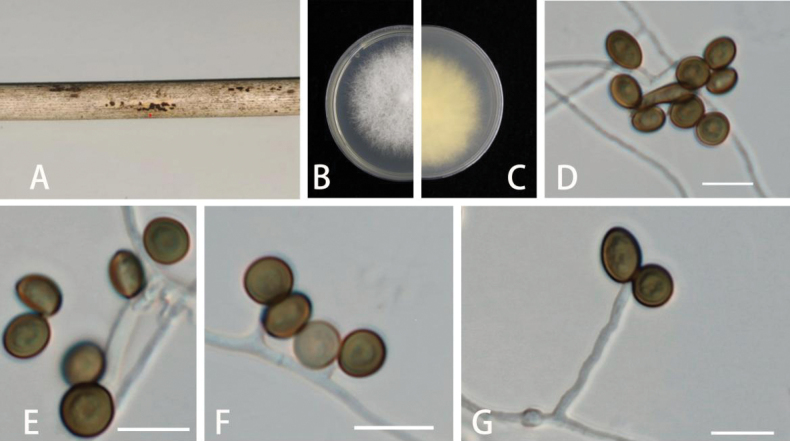
*Apiosporaarundinis* (GUCC 6.1) **A** dead bamboo branch with fungi **B, C** above and below view of the colony on PDA**D, E** conidia **F, G** conidiophores and conidiogenous cells. Scale bars: 10 µm (**D–G**).

##### Specimens examined.

China • Guizhou Province: Guiyang City, on diseased bamboo branch, 5 June 2022, K.M. Yu, living cultures: GUCC 6.1. and GUCC 6.2.

##### Notes.

In the present study, two new isolates (GUCC 6.1 and GUCC 6.2) clustered together with *Ap.arundinis* (CBS 133509) with high-support values (ML/BI = 98/1) in the multi-locus phylogenetic tree (Fig. 1). Morphologically, GUCC 6.1 and GUCC 6.2 have similar conidiophores, conidiogenous cells, and conidia to *Ap.arundinis* (Li et al. 2023). *Ap.arundinis* was found on various plants, including *Phyllostachyspraecox*, *Castaneamollissima*, and *Brunfelsiabrasiliensis* in China (Chen et al. 2014; Liao et al. 2022; Li et al. 2023). The conidia sizes of our collection (6–14 × 4–7 µm) larger than Chen et al. (2014) (5–7 × 2–4 µm) and Liao et al. (2022) (4.5–7.4 × 3.3–4.4 µm). Comparing with the description from Li et al. (2023) (6.4–10.4 × 5.2–8.3 µm), they have similar sizes, but the conidia in this study are slenderer and more elongated. Combining phylogenetic tree and morphology, these strains were identified as *Ap.arundinis*.

#### ﻿ Apiospora bambusicaulis

Taxon classificationFungiAmphisphaerialesApiosporaceae

K.M. Yu & Y.L. Jiang sp. nov.

1CD191E7-5C7E-539F-A0FF-EC9DFB87974B

854662

Fig. 3

##### Type.

China • Guizhou Province, Guiyang City, on diseased bamboo branch, 5 June 2022, K.M. Yu, holotype: HGUP 17.41, other living culture: GUCC 17.42.

##### Etymology.

Name refers to the host plant, meaning of bamboo stem, from which this fungus was isolated.

##### Description.

***Asexual morph***: On WA, hyphae smooth, branched, septate, hyaline to brown, 1–3.5 µm diam (n = 30). ***Conidiophores*** cylindrical, septate, straight to flexuous, sometimes reduced to conidiogenous cells. ***Conidiogenous cells*** globose to subglobose, erect, blastic, aggregated in clusters on hyphae, hyaline to pale brown, smooth, branched, 1.5–3.5 × 2–13.5 µm (x = 2.5 × 8 µm, n = 30). ***Conidia*** globose, sub-globose to ovate, lenticular, with a longitudinal germ slit over the entire length, brown to dark brown, smooth, 4.5–6 × 5–6 µm (x = 5.5 × 5.5 µm, n = 30). ***Sexual morph***: Not observed.

**Figure 3.**
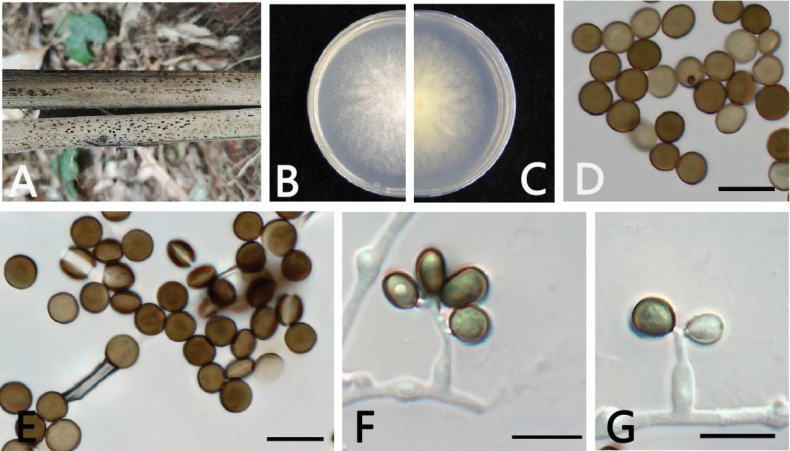
*Apiosporabambusicaulis* (GUCC 17.41, ex-type culture) **A** dead bamboo branch with fungi **B, C** above and below view of the colony on PDA**D, E** conidia **F, G** conidiogenous cells giving rise to conidia. Scale bars: 10 µm (**D–G**).

##### Culture characteristics.

***Colonies*** on PDA attaining 5 cm diam. after 4 days at 25 °C, circular, flat, radiating outwards, irregular edge, diffuse yellow pigment, the mycelia white to gray, floccose, cottony, reverse yellow.

##### Additional specimen examined.

China • Guizhou Province: Huaxi District, on diseased branch of bamboo, June 2023, K.M. Yu, HGUP 17.41, ex-paratype living culture; living cultures GUCC 17.41, GUCC 17.42.

##### Notes.

The phylogenetic tree indicated that *Ap.bambusicaulis* is closely related to a clade comprising *Ap.thailandica*, *Ap.jinanensis*, and *Ap.italica* with high support values (ML/BI = 99/1) in the multi-locus phylogenetic tree (Fig. 1). *Ap.bambusicaulis* differs from *Ap.thailandica* (Table 4) by 18 nucleotides (13/518 in ITS, 2/804 in LSU, 2/374 in *TEF1-α*, and 1/265 in *TUB2*), from *Ap.italica* by 58 nucleotides (17/518 in ITS, 4/799 in LSU, 26/377 in *TEF1-α*, and 11/266 in *TUB2*), and from *Ap.jinanensis* by 28 nucleotides (3/573 in ITS, 3/821 in LSU, 12/438 in *TEF1-α*, and 12/464 in *TUB2*). Morphologically, it differs from *Ap.thailandica*, *Ap.italica*, and *Ap.jinanensis* in conidia (brown, smooth, globose, 4.5–6 × 5–6 µm (x = 5.5 × 5.5 µm, n = 30) in *Ap.bambusicaulis* vs. globose, subglobose to lenticular, 5–9 × 5–8 μm in *Ap.thailandica* vs. brown, smooth, globose, (3–) 4–7 (–9) × (1.5–) 2–3 (–5.0) μm in *Ap.italica* vs. brown to dark brown, smooth to finely roughened 5.7–6.9 × 5.2–6.7 μm in *Ap.jinanensis* (Table 3), and base pair differences. Therefore, *Ap.bambusicaulis* is described as a new species, based on phylogeny and morphological comparison.

**Table 3.** Morphology of new *Apiospora* species and phylogenetic related species.

Species	Isolation	Source country	Conidia in surface view		References
			Shape	Diam (μm)	
*Apiosporabambusilentiginis*	Poaceae	China	globose, subglobose to ovate, lenticular	6–8.5 × 4.5–8	This study
*Ap.guizhouensis*	air in karst cave	China	globose or subglobose, occasionally elongated to ellipsoidal	5.0–7.5 × 4.0–7.0 µm (x¯ = 6.1 ± 0.5 × 5.5 ± 0.6	Wang et al. 2018
*Ap.sacchari*	soil	Netherlands	brown, smooth, granular, globose	(6–)7(–8)	Pintos and Alvarado 2018
*Ap.bambusicaulis*	Poaceae	China	globose, subglobose to ovate, lenticular,	4.5–6 × 5–6	This study
*Ap.italica*	Poaceae	Italy	brown, smooth, globose	(3.0–) 4.0–7.0 (–9.0) × (1.5–) 2.0–3.0 (–5.0)	Pintos et al. 2019
*Ap.jinanensis*	Poaceae	China	brown to dark brown, smooth to finely, roughened, globose, subglobose to lenticular,	5.7–6.9 × 5.2–6.7 μm, with a mean ± SD of 6.3 ± 0.3 × 5.6 ± 0.3	Ai et al. 2024
*Ap.thailandica*	Poaceae	Thailand	globose, occasionally elongated	5.0–9.0 × 5.0–8.0	Dai et al. 2017
*Ap.bambusirimae*	Poaceae	China	globose, subglobose to ovate	9.0–20 × 5–15.5	This study
*Ap.neogarethjonesii*	Poaceae	China	globose	20–35 × 15–30	Hyde et al. 2020
*Ap.setostroma*	Poaceae	China	obovoid, septate	18–20 × 15–19	Pintos and Alvarado 2021

**Table 4.** Comparison of DNA base differences between *Apiosporabambusicaulis* and related species.

Taxa	Loci	Nucleotide differences without gaps	Rates of base pair differences
*Apiosporaitalica*	ITS	3/571(51,119,460)	0.53%
	LSU	3/837(32,428,457)	0.36%
	*TEF*	30/464(30,34,41,55,106,120,164,166,189,224,233,241)	6.52%
	*TUB*	12/467(57,61,78,103,105,154,172,259,251,273,294,402)	2.57%
*Ap.thailandica*	ITS	9/430(54,96,120,375,402,405,417,430,445)	2.10%
	LSU	5/874(28,33,456,457)	0.57%
*Ap.jinanenisis*	ITS	3/573(101,445,523)	0.52%
		TUB:12/464(413,447,459,464,470,560,561,562,566,583,604)	
	LSU	3/821(392,393,780)	0.37%
		TEF:12/438(12,41,42,44,46,152,171,172,176)	
	TEF	12/438(12,41,42,44,46,152,171,172,176)	2.74%
	TUB	12/464(413,447,459,464,470,560,561,562,566,583,604)	2.56%

#### ﻿ Apiospora bambusilentiginis

Taxon classificationFungiAmphisphaerialesApiosporaceae

K.M. Yu & Y.L. Jiang sp. nov.

093DB8A5-6930-52F1-ABB6-23BAD74A9576

854667

Fig. 4

##### Type.

China • Guizhou Province: Guiyang City, on diseased stems of bamboo, 6 June 2023, 5 June 2022, K.M. Yu, holotype HGUP 18.51; ex-type culture GUCC 18.51).

##### Etymology.

The word bambusilentiginis originated from “bambusaceae,” referring to the host plant, and “speckle,” referring to cracks caused on bamboo stems, from which this fungus was isolated.

##### Description.

***Asexual morph***: On WA, hyphae smooth, branched, septate, 1–5.5 µm diam (n = 30). ***Conidiophores*** cylindrical, septate, flexuous, sometimes reduced to conidiogenous cells. ***Conidiogenous cells*** smooth, globose to subglobose, 1–5 × 1–2.5 μm (x̄ = 1.6 × 1.5 µm, n = 30). ***Conidia*** globose, subglobose to ovate, lenticular, with a longitudinal germ slit over the entire length, brown to dark brown, smooth, 6–8.5 × 4.5–8 µm (x̄ = 7.5 × 7.5 µm, n = 30). ***Sexual morph***: Not observed.

**Figure 4.**
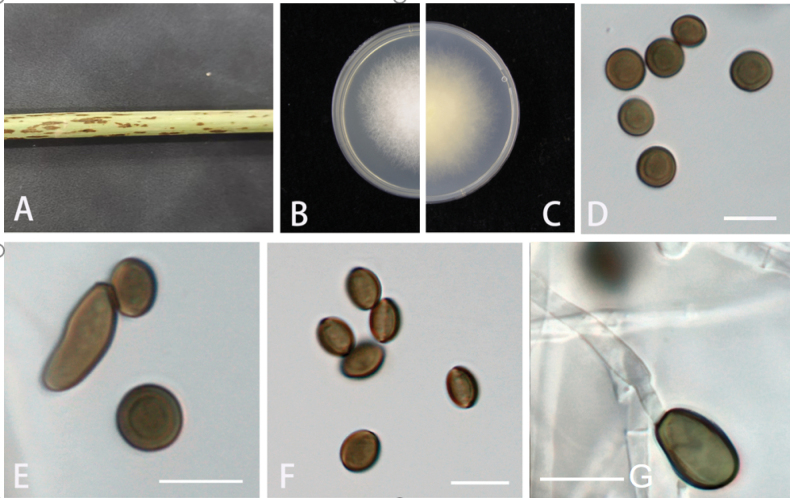
*Apiosporabambusilentiginis* (GUCC 18.51, holotype) **A** dead bamboo branch with fungi **B, C** above and below view of the colony on PDA**D-F**, conidia **G** conidiogenous cells giving rise to conidia. Scale bars: 10 µm (**D–G**).

##### Culture characteristics.

***Colonies*** on PDA attaining 5 cm diam. after 5 days at 25 °C, circular, flat, radiating outwards, irregular edge, diffuse yellow pigment, the surface of the culture medium is covered with aerial mycelia, mycelia white, reverse faint yellow.

##### Additional specimen examined.

China • Guizhou Province: Huaxi District, on diseased branch of bamboo, June 2023, K.M. Yu, HGUP 18.51, ex-paratype living culture; living cultures GUCC 18.51, GUCC 18.52

##### Notes.

The phylogenetic analysis showed that *Apiosporabambusilentiginis* is closely related to *Ap.guizhouensis* and *Ap.sacchari* (Fig. 1). They differ in distinct morphological characters (Table 3) and nucleotide differences (Table 5). *Apiosporabambusilentiginis* differs from *Ap.guizhouensis* by 18 nucleotides (8/580 in ITS, 8/442 in *TEF1-α*, and 2/440 in *TUB2*) and *Ap.sacchari* by 42 nucleotides (35/583 in ITS, 1/440 in *TEF1-α*, and 6/483 in *TUB2*). Morphologically, it differs from *Ap.guiyangensis* and *Ap.sacchari* in its conidia. The conidia of *Apiosporabambusilentiginis* are globose, subglobose to ovate, lenticular, while the conidia of *Ap.guizhouensis* are guttulate, globose to ellipsoid. In addition, comparing with *Ap.guizhouensis* (5.0–7.5 × 4.0–7.0 μm), the conidia of *Apiosporabambusilentiginis* (6–8.5 × 4.5–8(x = 7.5 × 7.5 µm, n = 30) show larger sizes (Crous and Groenewald 2013; Wang et al. 2018).

**Table 5.** DNA base differences comparing *Apiosporabambusilentiginis* sequences and sequences from related species.

Taxa	Loci	Nucleotide differences without gaps	Rates of base pair differences
*Apiosporaguizhouensis*	ITS	8/580(13,401,537,549,550,560)	1.38%
	TEF	8/442(17,24,132,146,190,322,324,325)	1.80%
	TUB	2/440(296,408	0.05%
*Ap.sacchari*	ITS	35/583(48,51,5,55,56,60,94,98,99,125,136,346.358,366,374,377,391,414,419,432,344,350,363,365,367,368,468,469,471,477,481,494,514,520,525)	6.00%
	TEF	1/440	0.23%
	TUB	6/483(63,116,269,352)	1.24%

#### ﻿ Apiospora bambusirimae

Taxon classificationFungiAmphisphaerialesApiosporaceae

K.M. Yu & Y.L. Jiang sp. nov.

D1121EC3-EC1E-54D7-BCD4-D1BE9F164915

854664

Fig. 5

##### Type.

China • Guizhou Province, Guiyang City, on diseased bamboo branch, 5 June 2022, K.M. Yu, holotype: HGUP 12.51; ex-type culture: GUCC 12.51. other living culture: GUCC 12.52

##### Etymology.

Name bambusirimae originated from “bambusaceae,” referring to the host plant, meaning bamboo-crack, referring to cracks caused on bamboo stems, from which this fungus was isolated.

##### Description.

***Asexual morph***: On WA, hyphae smooth, branched, septate, hyaline to brown, 1.5–3 µm diam (n = 30). ***Conidiophores*** basauxic, cylindrical, smooth, septate, straight or flexuous, hyaline to brown, sometimes reduced to conidiogenous cells. ***Conidiophore mother cells*** arising from the stroma, lageniform to ampuliform, hyaline to brown, 13.5–28 × 2–5.5 µm (x = 20 × 4 µm, n = 30). ***Conidia*** globose, subglobose to ovate, with a longitudinal germ slit over the entire length, with granular depositions, brown to dark brown, smooth, 9–20 × 5–15.5 µm (x = 20.5 × 13 µm, n = 30). ***Sexual morph***: Not observed.

**Figure 5.**
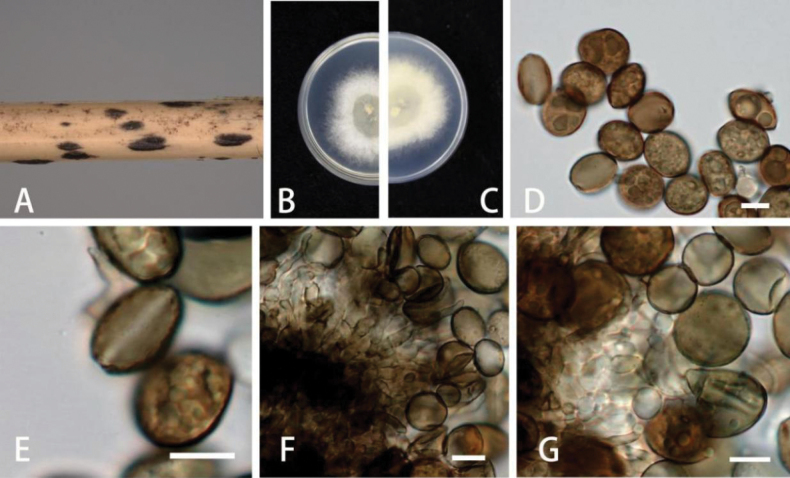
*Apiosporabambusirimae***A** dead bamboo branch with fungi **B, C** above and below view of the colony on PDA**D, E** conidia **F, G** conidiogenous C rise to conidia. Scale bars: 10 µm (**D–G**).

##### Culture characteristics.

***Colonies*** on PDA attaining 3.5 cm diam. after 3 days at 25 °C, circular, flat, radiating outwards, irregular edges, diffuse yellow pigment, the mycelia white and floccose, cottony, reverse pale yellow.

##### Additional specimen examined.

China • Guizhou Province: Huaxi District, on diseased branch of bamboo, June 2023, K.M. Yu, HGUP 12.51, ex-paratype living culture; living cultures GUCC 12.51, GUCC 12.52.

##### Notes.

*Ap.bambusirimae* is genetically close to *Ap.neogarethjonesii* and *Ap.setostroma* (Fig. 1), but the conidia of *Ap.bambusirimae* are 9–20 × 5–15.5 µm (x̄ = 20.5 × 13 µm, n = 30), shorter and narrower than those of *Ap.neogarethjonesii* and *Ap.setostroma*, which are 20–35 × 15–30 µm and 18–20 × 15–19 µm. We compared the new species with phylogenetically related taxa, based on morphological differences (Table 3) and base pair differences (Table 6).

**Table 6.** DNA base differences comparing *Apiosporabambusirimae* sequences and sequences from related species.

Taxa	Loci	Nucleotides difference without gaps	Rates of base pair differences
*Apiosporaneogarethjonesii*	ITS	7/430(36,327,368,383,389,394,395,453	1.63%
	LSU	7/881(101,356,584,626,635,654,659)	0.79%
*Ap.setostroma*	ITS	8/430(37,327,368,381,387,392,393,451)	1.86%
	LSU	8/837(100,356,369,384,626,635,654,659)	0.96%

#### ﻿ Apiospora lophatheri

Taxon classificationFungiAmphisphaerialesApiosporaceae

S.J. Li & C.M. Tian, MycoKeys 99: 297–317 (2023)

070989BD-6B36-54B8-A9A4-74DBBAEB064D

Fig. 6

##### Description.

***Asexual morph***: On WA, hyphae of smooth, hyaline, branched, septate, 1–5 µm diam. hyphae (n = 30). ***Conidiogenous cells*** subglobose to ampulliform, doliiform, clavate, erect, aggregated in clusters on hyphae, smooth, branched, 3–14 × 1.5–3.5 µm (x = 8.5 × 6 µm, n = 30). ***Conidia*** globose to subglobose, occasionally elongated to ellipsoidal, lenticular, with a longitudinal germ slit, brown to dark brown, smooth, 4–6.5 × 3–6 µm (x = 5.5 × 5 µm, n = 30). ***Sexual morph***: Not observed.

##### Culture characteristics.

***Colonies*** on PDA attaining 5 cm diam. after 4 days at 25 °C, thick, dense, margin undulate and irregular, diffuse yellow pigment, surface with patches of grey aerial mycelia, and reverse yellow.

##### Specimens examined.

China • Guizhou Province, Guiyang City, on diseased bamboo branch, 5 June 2022, K.M. Yu, living cultures: GUCC 21.11. and GUCC 21.12.

##### Notes.

*Ap.lophatheri* was isolated from *Lophatherumgracile* in China (Li et al. 2023). Phylogenetic analyses indicated that the two new isolates (GUCC 21.11 and GUCC 21.12) clustered together with *Ap.lophatheri* with high support values (ML/BI = 100/0.99) (Fig. 1). Morphologically, our collection has similar conidia to *Ap.lophatheri* (4–6.5 × 3–6 µm (x = 5.5 × 5 µm vs. 5.1–8.9 × 4.6–7.7 µm) (Li et al. 2023). Thus, these isolates were identified as *Ap.lophatheri*, and bamboo as a new host record.

**Figure 6.**
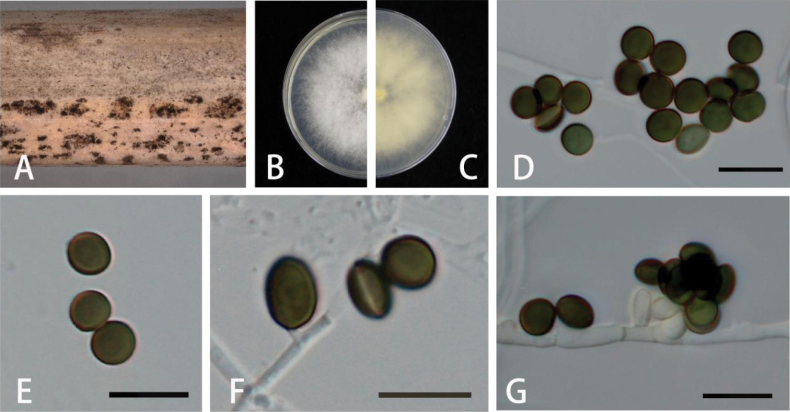
*Apiosporalophatheri* (GUCC 21.11) **A** dead bamboo branch with fungi **B, C** above and below view of the colony on PDA**D-F** conidia **F, G** conidiogenous cells giving rise to conidia. Scale bars: 10 µm (**D–G**).

## ﻿Discussion

After undergoing significant changes, the classification of *Apiospora* has now been proposed in Apiosporaceae within Amphisphaeriales and together with *Arthrinium* and *Neoarthrinium* as three distinct lineages (Hyde et al. 1998; Crous and Groenewald 2013; Wang et al. 2018; Pintos et al. 2019; Pintos and Alvarado 2021; Jiang et al. 2022). Morphologically, *Apiospora*, *Arthrinium*, and *Neoarthrinium* are similar in their same basauxic conidiogenesis, but the conidiophores of some *Arthrinium* and *Neoarthrinium* species have thick blackish septa, which are infrequently observed in *Apiospora* (Ellis 1965; Wang et al. 2018; Pintos and Alvarado 2021).

The *Apiospora* species are widely distributed around the world; there are over 90 accepted species (Table 2) and approximately 50 in China. Asia has been found to have the highest diversity of *Apiospora*, which is probably the result of the rich diversity of bamboo species, especially in China, which has the richest variety of bamboo density (Lobovikov et al. 2007). *Apiospora* has been found in provinces including Guizhou, Sichuan, Hainan, Jiangxi, Yunnan, Guangxi, Shandong, etc., in China (Zhang and Lu 2023; Li et al. 2023; Yan and Zhang 2024; Ai et al. 2024). Although *Apiospora* is mainly collected in the Poaceae family, some others from the Lauraceae, Primulaceae, Theaceae, and Solanaceae plant families, *Dichotomanthustristaniaecarpa* and *Corylusyunnanensis* (Wang et al. 2018; Jiang et al. 2018; Jiang and Tian 2021; Pintos and Alvarado 2021; Li et al. 2023).

In this study, three new *Apiospora* species (*Ap.bambusicaulis*, *Ap.bambusirimae*, and *Ap.bambusilentiginis*) and two recognized species (*Ap.arundinis* and *Ap.lophatheri*) collected from bamboos in Guiyang City of Guizhou Province are introduced. This is consistent with previous studies (Wang et al. 2018; Jiang and Tian 2021; Liao et al. 2022; Li et al. 2023), where all of the *Apiospora* species we collected come from the Poaceae located in subtropical regions (on bamboo in Guizhou, China). Therefore, the *Apiospora* identification has been improved through research combined on morphological and molecular characteristics, host communities, and ecological distribution.

## ﻿Conclusion

The preference of *Apiospora* for hosts and habitats is helpful for the study of this genus. China has abundant biological resources, with bamboo as a representative species widely distributed here. Therefore, a comprehensive investigation of *Apiospora* species in China is necessary. The differences in lifestyle and host range diversity of *Apiospora* and the speed of discovery suggest that the number of species may continue to increase in the future. In addition, the morphological differences of *Apiospora* may be related to the hosts. Investigation and systematic classification of *Apiospora* are expected to discover more fungal resources of this genus, which is of great significance for this genus of fungi.

## Supplementary Material

XML Treatment for Apiospora arundinis

XML Treatment for Apiospora bambusicaulis

XML Treatment for Apiospora bambusilentiginis

XML Treatment for Apiospora bambusirimae

XML Treatment for Apiospora lophatheri

## ﻿Additional information

### Author ORCIDs

Kunmin Yu https://orcid.org/0009-0009-2433-392X

Hong Zhang https://orcid.org/0000-0003-1894-8169

Kexin Cheng https://orcid.org/0009-0008-5478-5161

Yulan Jiang https://orcid.org/0000-0002-2444-3506

### Data availability

All of the data that support the findings of this study are available in the main text.
